# Small Molecules Greatly Improve Conversion of Human-Induced Pluripotent Stem Cells to the Neuronal Lineage

**DOI:** 10.1155/2012/140427

**Published:** 2012-04-10

**Authors:** Sally K. Mak, Y. Anne Huang, Shifteh Iranmanesh, Malini Vangipuram, Ramya Sundararajan, Loan Nguyen, J. William Langston, Birgitt Schüle

**Affiliations:** Basic Research Department, The Parkinson's Institute, 675 Almanor Ave, Sunnyvale, CA 94085, USA

## Abstract

Efficient *in vitro* differentiation into specific cell types is more important than ever after the breakthrough in nuclear reprogramming of somatic cells and its potential for disease modeling and drug screening. Key success factors for neuronal differentiation are the yield of desired neuronal marker expression, reproducibility, length, and cost. Three main neuronal differentiation approaches are stromal-induced neuronal differentiation, embryoid body (EB) differentiation, and direct neuronal differentiation. Here, we describe our neurodifferentiation protocol using small molecules that very efficiently promote neural induction in a 5-stage EB protocol from six induced pluripotent stem cells (iPSC) lines from patients with Parkinson's disease and controls. This protocol generates neural precursors using Dorsomorphin and SB431542 and further maturation into dopaminergic neurons by replacing sonic hedgehog with purmorphamine or smoothened agonist. The advantage of this approach is that all patient-specific iPSC lines tested in this study were successfully and consistently coaxed into the neural lineage.

## 1. Introduction

The advent of nuclear reprogramming of somatic cells into induced pluripotent stem cells (iPSCs) for *in vitro* disease modeling also accelerated the field of differentiation into specialized cell types. Differentiation into specific lineages had its primary place to provide a resource for cell replacement therapies [1]. These specialized differentiated cells were in general derived from a small number of “approved” human embryonic stem cell lines [2, 3].

 Patient-specific iPSC-derived differentiated cells have now become an attractive tool to study disease mechanisms on a human background and are a vanguard into a new era of science and potentially personalized medicine. In particular for monogenic forms of disease, patient-derived iPSCs have already been shown to recapitulate known disease mechanisms, as shown in spinal muscular atrophy [4], fragile X syndrome [5], progeria syndrome [6], and several genetic forms of Parkinson disease (PD) like LRRK2 [7], PINK1 [8], SNCA [9], and GBA [10]. This novel approach of disease modeling becomes very attractive for drug screening and discovery [11, 12].

 One of the challenges is to differentiate these patient-derived iPSCs into the desired specialized cell type of interest. For neuronal differentiation, there were three main approaches developed in the last decade to derive dopaminergic neurons [13–15]. The first method is stromal-induced neuronal differentiation, termed stromal cell-derived inducing activity (SDIA) [16, 17]. The concept is that mouse stromal cells such as PA6 or MS5 or midbrain astrocytes were used to coax the regionalization of stem cells. The disadvantage of this method is the variability of stromal cells and unknown factors; furthermore, this protocol is overall lengthy and takes about 40–60 days *in vitro*. The second main approach is embryoid body (EB)/neurosphere-mediated differentiation [18, 19], caveats are clonal expansion of subgroup of cells and potential forebrain specification. However, the usefulness of neuronal precursors (NPCs) is that they can be expanded, cryopreserved, and be a starting pool for final maturation. NPCs are also important for scientific questions of developmental phenotypes related to disease. The third approach of direct neuronal differentiation utilizes high-density monolayer ESC/iPSC cultures via floor-plate formation which gives the promise of shortening the time for neuronal development while reaching high differentiation efficiency of midbrain dopaminergic neurons [15] and show excellent survival and functional benefit which gives hope for regenerative therapies in Parkinson's disease [20].

 Here, we describe a 5-stage EB differentiation approach using small molecules to enhance neural induction consistently in patient and control iPSC lines. In addition, we substituted sonic hedgehog (Shh) with purmorphamine (Pur) and/or smoothened agonist (SAG) to reduce cost for the final maturation and have shown comparable results between Shh and these small molecules. We have made considerable progress in consistency and reproducibility of this process; however, there is still a challenge of improving the overall yield of region-specific dopaminergic neurons.

## 2. Materials and Methods

### 2.1. Skin Biopsies of Patient and Control Subjects

Skin punch biopsies (4-mm circular) were taken from all individuals employing a standard punch biopsy [21]. All biopsies were taken from the upper inner arm, an area that is mostly unexposed to direct sunlight. We used a standard skin explant culture technique by cutting the biopsy tissue into 12–15 pieces and placed 2-3 pieces into one well of a gelatinized 6-well plate in 1 mL of high-glucose DMEM, 20% fetal bovine serum, 1x nonessential aminoacids (NEAA), 1x penicillin streptomycin (P/S), 1x L-glutamine (Glu) (all were purchased from Invitrogen, Carlsbad, CA). Outgrowth of keratinocytes was first noted 2–5 days after plating. Cells were expanded using standard tissue culture techniques, cells were passaged upon confluency using trypsin/EDTA (Invitrogen) and 15–20 million cells. Miocells were cryopreserved for banking. This study and protocol had Institutional Review Board approval and all subjects gave written informed consent for this study. Clinical information on the patients is provided in Supplementary Table 1 (see supplementary materials available at doi:10.1155/2012/140427).

### 2.2. Generation of iPSC

All iPSCs were derived using a retroviral system to deliver four genes encoding OCT4, KLF4, SOX2 and cMYC (from Addgene plasmids 17217, 17218, 17219, 17220, http://www.addgene.org/) using published protocols [22, 23]. All lines have been characterized for pluripotency, differentiation potential into three germ layers, are karyotypically normal and genotype-match the parental fibroblasts, (see Supplementary Table 2). IPSC line 1761 was previously described and characterized in Nguyen et al. 2011 [7].

### 2.3. iPSC Maintenance and Propagation

iPSCs were cultured and maintained on mitomycin C inactivated mouse embryonic fibroblasts (iMEF) (EMD Millipore Cat. No. PMEF-CF) in hESC media containing DMEM/F12, 20% knockout serum replacement (Invitrogen, Cat. No. 10828028), 1x NEAA, 1x P/S, 0.1%  *β*-Mercaptoethanol (Invitrogen, Cat. No. 21985023), 0.5x L-Glu and 6 ng/mL of basic fibroblast growth factor (FGF2) (Cat. No. 233-FB, R&D Systems, Minneapolis, MN). Cells were split every week manually without enzymatic treatment.

### 2.4. Generation and Maintenance of Neural Progenitor Cells (NPCs)

To derive NPCs, iPSC colonies were harvested using 1 mg/mL of collagenase IV (Invitrogen, Cat. No. 174104019). After about 1 hr, when all colonies lifted up completely from culture dish, colonies were transferred to 10 cm bacterial petri dishes (BD Bioscience, Bedford, MA). Forming embryoid bodies (EB) was cultured in suspension with agitation on rocker (Rocker II Model 260350, Boekel Scientific, Feasterville, PA) for 4 days in EB media, which consisted of hESC media minus FGF2 with or without 5 *μ*M dorsomorphin (Dor) (Sigma, St Louis, MO, Cat. No. P5499) and 10 *μ*M SB431542 (SB) (Tocris Bioscience, Ellisville, MO, Cat. No. 1614).

 Next, EBs were cultured for additional 2-3 days with agitation in neural induction media (NIM) consisting of DMEM/F12 (Invitrogen, Cat. No. 12500, powder form), 1x NEAA, 0.5x L-Glu, and freshly made and sterile filtered N2, which contained 1.55 g/L glucose (Sigma, Cat. No. G7021), 2 g/L sodium bicarbonate (Sigma, Cat. No. S5761), 100 *μ*M putrescine (Sigma, Cat. No. P5780), 30 nM sodium selenite (Sigma, Cat. No. S9133), 20 nM progesterone (Sigma, Cat. No. P8783), 0.1 mg/mL transferrin (Sigma, Cat. No. T0665), 0.025 mg/mL insulin (Sigma, Cat. No. I6634) and FGF2 (20 ng/mL). Cell culture plates were coated with Geltrex (Invitrogen, Cat. No. 12760021), media changed every day. Neural rosettes were formed in 2–5 days in adherent culture.

 To obtain a pure population of NPCs, rosettes were manually isolated using No. 15 scalpel cutting in squares with distance to edges of colonies (Figure 3(F)). Dissected pieces of rosettes were lifted using a pipette, replated onto Geltrex-coated culture dishes and maintained in neural progenitor cell media (NPC media) containing neurobasal media (Invitrogen, Cat. No. 21103049), 1x NEAA, 1x L-Glu, 1x P/S, 1x B27 supplement (Invitrogen, Cat. No. 17504044), and FGF2 (20 ng/mL). Manual isolation of rosettes as described above was repeated once to obtain more pure population of NPCs. Approximately 5–10 pieces of rosettes were dissociated into single cells using Accutase (MP Biomedicals, Solon, OH, Cat. No. 0910004). Cells were treated with Accutase for 2–5 minutes until cells became round in shape, then the cells were collected, centrifuged, resuspended in NPC media, and plated onto one 96-well coated with Geltrex and cultured at 37°C and 5% CO_2_. When confluent, NPCs were split at a ratio of 1 : 2 in single wells with larger surface area such as 48-well, 24-well, 12-well, and so forth in NPC media.

### 2.5. NPC Enrichment Using Anti PSA-NCAM Microbeads

For magnetic bead sorting, NPCs were treated with Accutase, collected, and passed through 30 *μ*m nylon mesh (pre-separation filters, 30 *μ*m, Miltenyi Biotec Auburn, CA, Cat. No. 130-041-407). The total cell number was approximately 10^7^. Cell suspension was centrifuged at 300 ×g for 10 minutes. Supernatant was aspirated completely and cell pellet was resuspended in 60 *μ*L of buffer (1x PBS, 2 mM EDTA (Ambion Inc, Austin, TX, Cat. No. AM9260G) and 0.5% albumin from bovine serum (BSA) (Sigma, Cat. No. A3294). Cells were mixed well and incubated for 10 minutes in the refrigerator (2−8°C). Then, 20 *μ*L of anti-PSA-NCAM microbeads (Miltenyi Biotech, Auburn, CA, Cat. No. 130-092-966) were added to the mixture, mixed well with pipetting up and down, and incubated for 15 minutes in the refrigerator (2−8°C). Cells were washed by adding 2 mL of buffer and centrifuged at 300 ×g for 10 minutes. Supernatant was aspirated completely and cell pellet was resuspended up to 10^8^ cells in 500 *μ*L of buffer. A MS column (Miltenyi Biotec, Cat. No. 130-042-201) was placed in the magnetic field of a miniMACS separator (Miltenyi Biotec, Cat. No. 130-042-102), rinsed with 500 *μ*L of buffer three times. Cell suspension was applied onto the column. The column was washed with 500 *μ*L of buffer three times again. New buffer was added when the column reservoir was empty. The column was removed from the separator and placed on a 15 mL BD Falcon conical tube (BD Bioscience, 352097). One mL of buffer was added onto the column and magnetically labeled cells were flushed out by firmly pushing the plunger into the column. The eluted fraction was directly enriched over a second column and the magnetic separation procedure was repeated once by using a new MS column. One mL of NPC media was added onto the column to flush out the magnetically labeled cells. Then another 1 mL of NPC media was added and cell suspension was transferred to a 35 mm Geltrex-coated culture dish.

### 2.6. Dopaminergic Differentiation of NPCs

Dopaminergic differentiation was initiated by culturing NPCs on Geltrex-coated culture dishes or glass coverslips (Fisher Scientific, Pittsburg, PA, Cat. No. 12-545-80-12CIR-1) coated with poly-L-ornithine (20 *μ*g/mL) (Sigma, Cat. No. P4957) at 37°C for 4 hrs and laminin (Sigma Cat. No. L2020) (20 *μ*g/mL) at 4°C overnight.

 Dopaminergic differentiation in defined media was initiated by culturing ~0.5 × 10^6^ NPCs (in 35 mm culture dish) in DA1 media for 10 days in Neurobasal media supplemented with 1x NEAA, 1x L-Glu, 1x P/S, 1x B27, FGF8b (100 ng/mL) (R&D Systems, Cat. No. 423-F8) and tested with either 2 *μ*M Purmorphamine (Pur) (EMD Chemicals, Cat. No. 540220), Gibbstown, NJ), 0.4 mM SAG (Enzo Life Sciences, Farmingdale, NY, Cat. No. ALX-270-426-M001) or 200 ng/mL Sonic Hedgehog, C24II (Shh) (R&D Systems, Cat. No. 1845-SH). During these 10 days, when cells grew confluent, they were passaged with Accutase as described above and replated onto a Geltrex-coated plates at cell density of ~80%. Lower density led to cell death.

 Final maturation into dopaminergic neurons was carried out in DA2 media containing neurobasal media supplemented with 1x NEAA, 1x L-Glutamine, 1x B27 supplement, 1x Penicillin-Streptomycin, 20 ng/mL BDNF (R&D Systems, Cat. No. 248-BD), 20 ng/mL GDNF (R&D Systems, Cat. No. 212-GD), and 1 mM dibutyryl cAMP (Sigma, Cat. No. D0627) for 20–30 days. Cells were not continued to passage in DA2 media when the processes formed. Cells were analyzed on day 30 of the maturation process.

### 2.7. Quantitative PCR (qPCR)

EBs were collected on day 6 of EB formation before plating down for rosette formation. Total RNA was extracted using a RNeasy Micro kit (Qiagen, Valencia, CA) and 500 ng RNA was used for reverse-transcription into cDNA using the iScript cDNA Synthesis Kit (BioRad, Hercules, CA). Total reaction volume was 20 *μ*L; the resulting cDNA sample was diluted with 80 *μ*L of ultrapure water, and 5 *μ*L of the diluted cDNA sample was used as template for qPCR amplification. qPCR was performed using ABI PRISM 7000 Sequence Detection System (Applied Biosystems, Foster City, CA). All reactions were run in 20 *μ*L reactions volume using SYBR Green PCR Master Mix (Applied Biosystems) and 30 pmol of each primer. qPCR parameters were as follows: 2 min at 50°C; 10 min at 95°C; 40 cycles at 95°C for 15 sec, and at 60°C for 1 min. Data were collected at 60°C. All data were normalized to *β-actin* expression and plotted as fold changes over samples from EBs without Dor/SB. Primers of genes used in this study: Sox1 (5′-GAGATTCATCTCAGGATTGAGATTCTA-3′ and 5′-GGCCTACTGTAATCTTTTCTCCAC-3′); Nestin (5′-TGCGGGCTACTGAAAAGTTC-3′ and 5′-AGGCTGAGGGACATCTTGAG-3′); Brachyury (5′-AGGTACCCAACCCTGAGGA-3′ and 5′-GCAGGTGAGTTGTCAGAATAGGT-3′); GATA4 (5′-GTCATCTCACTACGGGCACA-3′ and 5′-CTTCAGGGCCGAGAGGAC-3′); Oct4 (5′-TGGGCTCGAGAAGGATGTG-3′ and 5′-GCATAGTCGCTGCTTGATCG-3′) and *β*-actin (5′-CTGAACCCCAAGGCCAAC-3′ and 5′-TAGCACAGCCTGGATAGCAA-3′).

### 2.8. Immunocytochemistry

EBs and NPCs were fixed with 4% paraformaldehyde (PFA) (Electron Microscopy Sciences, Hatfield, PA) at room temperature for 10 minutes. Neurons were fixed carefully by adding equal volume of 8% PFA into wells to equal volume of medium and incubated at room temperature for 10 minutes. Fixed cells were permeabilized with 0.3% Triton X-100 (Sigma, Cat. No. X100) for 5 minutes and were blocked in blocking buffer (5% normal goat serum, Vector Labs, Burlingame, CA) in 1x Phosphate buffered saline (PBS) (Sigma, Cat. No. P5493) and followed by incubation with the primary antibody at 4°C overnight in 5% normal goat serum and PBS. The following primary antibodies were used: Nestin (EMD Millipore, Billerica, MA, Cat. No. MAB5326), 1 : 200; Sox1 (EMD Millipore, Cat. No. AB15766), 1 : 100; Tyrosine Hydroxylase (TH) (PelFreez Biologicals, Rogers, AR, Cat. No. P40101-0), 1 : 300; Pax6 (Developmental Studies Hybridoma Bank, Iowa City, IA, Cat. No. Pax6), 1 : 20; neuronal class III *β*-Tubulin (TUJ1) (Covance, Princeton, NJ, Cat. No. MMS-435P), 1 : 500, and secondary antibodies were Alexa Fluor 488 Goat Anti-Mouse, Alexa Fluor 555 Goat Anti-Mouse, Alexa Fluor 488 Goat Anti-Rabbit, Alexa Fluor 555 Goat Anti-Rabbit (Invitrogen) at 1 : 300. Coverslips were mounted with Vectashield Mounting Medium with DAPI (Vector Labs). Fluorescent images were captured on an Eclipse Ti inverted fluorescence microscope (Nikon Instruments Inc, Melville, NY). Phase contrast images were taken with a Zeiss Axiovert 25 Inverted Microscope (Carl Zeiss AG, Oberkochen, Germany).

### 2.9. Stereological Analysis

Stereological analysis was performed using an Olympus BH2 microscope (Olympus, Center Valley, PA) with a motorized X-Y stage linked to a computer-assisted stereological system (Olympus America Inc.). This comprises a color video camera (CCD-Iris, Sony), a PC with a high-resolution SVGA monitor, a microcator (VRZ 401, Heidenhain), and Stereo Investigator (MBF Bioscience, Williston, VT). Immunostained coverslips were delineated at 4x magnification. From a random start position, a counting frame was superimposed on the image, and cells were systematically sampled using a 40x objective lens (Olympus), with DAPI stained nucleus used as the sampling unit. A minimum of 200 cells was sampled according to the rules of the optical dissector [24], and the coefficient of error for each stereological estimate was between 0.07 and 0.1 [25].

### 2.10. Flow Cytometry

After dopaminergic differentiation, cells were dissociated with TrypLE Express (Invitrogen, Cat. No. 12605-028) at 37°C for 5 minutes, washed with PBS, centrifuged, resuspended in PBS, and strained through a 70 *μ*m cell strainer (BD Biosciences, Cat No. 352350), centrifuged, resuspended, and fixed in 4% PFA in PBS at room temperature for 10 minutes. Then they were centrifuged, resuspended, and permeabilized with 0.3% saponin (Sigma, Cat. No. 47036), incubated with TUJ1 (Covance, 1 : 100) and TH (Pel Freez Biologicals, 1 : 100) on ice for 30 minutes and washed with washing buffer (PBS and 0.03% saponin) once. Then cells were incubated with APC-conjugated anti mouse IgG antibody (BD Biosciences, Cat. No. 550826) and PE-conjugated anti rabbit IgG antibody (BD Biosciences, Cat. No. 558416) on ice for 30 minutes, washed with washing buffer, and resuspended in PBS. All sorting procedures were carried out using BD Digital Vantage (BD Biosciences) with a 80 *μ*m nozzle. Data were analyzed by FlowJo flow cytometry software (Version 7.6.4, Tree Star Inc, Ashland, OR). We compared cell suspension of unstained NPCs and unstained differentiated cells as negative control to determine the threshold for detection of immunofluorescence. 

### 2.11. Statistical Analysis

**Figure 1:**
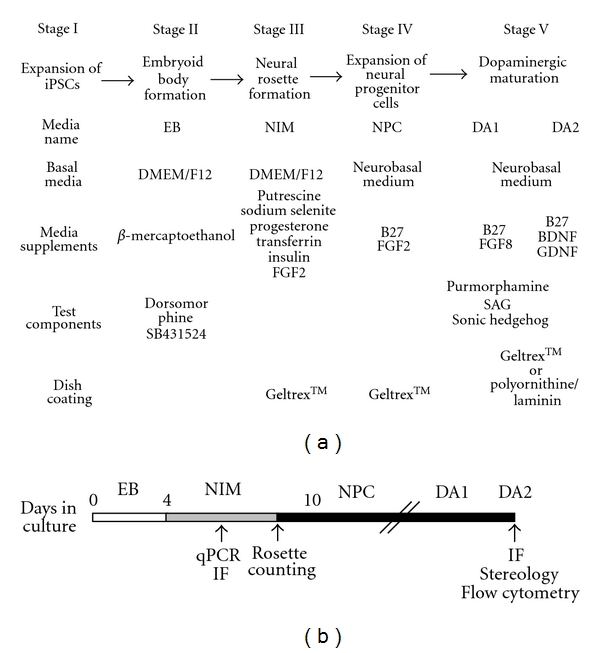
Schematic summary of the differentiation conditions used in the generation of dopaminergic neurons (a) Schematic diagram illustrating the different stages of NPC generation and dopaminergic neuronal differentiation. The abbreviations are. Dor/SB: Dorsomorphin and SB431542, EB: EB media, NIM: neural induction media, NPC: neural progenitor cell media, and DA1/DA2: medium for dopaminergic differentiation. (b) Timeline shows the medium used at different stages of NPCs and dopaminergic maturation and displays the sampling dates for performing gene expression studies, such as qPCR, marker characterization (immunofluorescence: IF), to analyze efficiency of rosette formation and for quantitative studies of TUJ1 and TH immunoreactivity using stereology and flow cytometry.

Statistical analysis was performed using GraphPad Prism (Version 4, GraphPad Software, San Diego, CA). Data were analyzed by one-way analysis of variance (ANOVA). Newman-Keuls post hoc analysis was employed when differences were observed in ANOVA testing (*P* < 0.05). Data were presented as the means + standard error of the mean (SEM). All results were derived from at least three independent experiments, except results of cell line 1679 in Figure 1 and flow cytometry data in Figure 6 were derived from two independent experiments.

## 3. Results and Discussion

### 3.1. Neuronal Differentiation Using a 5-Stage Embryoid Body Approach

The majority of published neuronal differentiation methods describe selected human embryonic stem cell (hESC) lines such as H9 or I6 and these protocols were optimized around these cell lines. Despite general reproducibility across multiple hESC lines, in patient-specific human iPSCs consistent reproducibility has not been demonstrated, posing a challenge for disease modeling and drug screening. [26–29]. One recent publication points towards specific markers such as miR-371-3 and FoxA2 that could predict a priori the differentiation potential of iPSCs or ESCs into the neuronal lineage, which can be relevant for downstream applications [30].

 Our goal was to develop a reliable protocol reproducible across various patient-specific iPSC lines. We tested a 5-stage protocol for neuronal dopaminergic differentiation that was originally introduced by Lee and Studer in mouse embryonic stem cells [18] and subsequently further developed [28, 29]. This protocol involves EB formation for four days, neural rosette formation, isolation of neural rosettes, and expansion and PSA-NCAM enrichment using magnetic bead sorting of neuroprogenitors. A final maturation stage utilizes FGF8 and sonic hedgehog (Shh) for the first ten days followed by BDNF, GDNF, B27, and dcAMP in Neurobasal media for another 20–50 days (Figure 1(a)). We reason that this 5-stage protocol generating EBs has several advantages in generating neural precursors that can be easily expanded without loss of differentiation potential [31]. Thus, this protocol is suitable for studying disease-mechanisms at the neuroprogenitor stage and maintaining potential for derivation of other CNS cell types [28].

 In a control iPSC line, EBs incubated for 4 days in EB media showed a similar result of neural rosette formation as described by Swistowski et al., 2009 [28] (data not shown). When we attempted to derive NPCs from additional iPSCs derived from controls and patients affected with PD we observed very little neural rosette formation. Furthermore, these rosettes were not expandable as NPCs.

 Small molecules have been reported to improve directing ESC/iPSCs into neural lineage [32, 33]. We tested a combination of small molecules: Dor and SB, both of which have been described for SMAD inhibition. The synergistic mode of action of inhibitors of SMAD signaling, Noggin and SB431542, has been reported to rapidly induce neural conversion of hESCs [15, 34]. Noggin, a bone morphogenetic protein (BMP) antagonist, and the small molecule Dor have similar activities which selectively inhibit the BMP type I receptors: ALK2, ALK3, and ALK6 and block SMAD1/5/8 phosphorylation [35]. SB has been shown to be a selective inhibitor of activin receptor-like kinase receptors ALK4, ALK5, and ALK7 [36].

**Figure 2:**
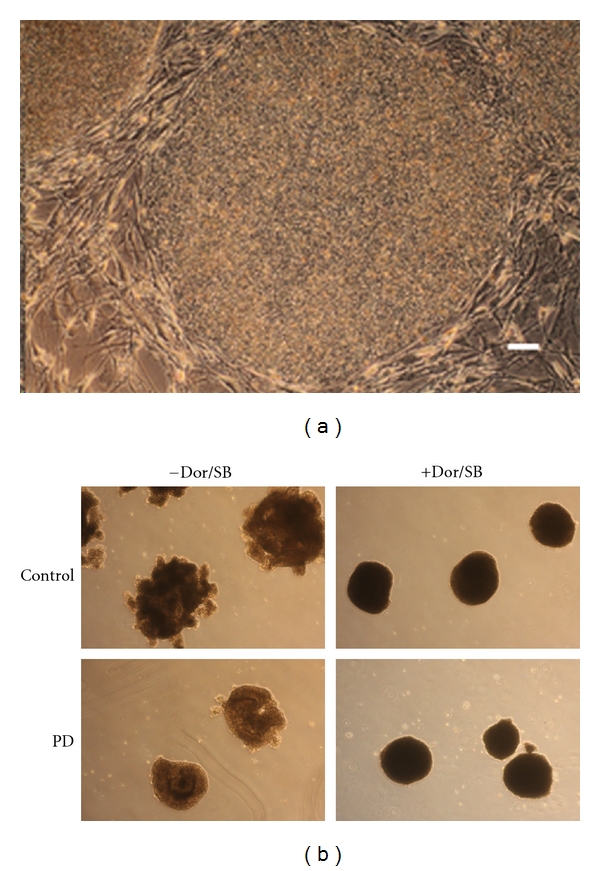
(a) Representative image of the quality of iPSC colonies used to produce EBs: distinct border with little to no differentiation. The recommended size for EB formation should be double the size as the depicted colony, approximately 2 mm in diameter. Scale bar 100 *μ*m (b) In-control and PD-specific cell lines (Control and PD), EBs were lacking that compact structure and round borders when cultured without Dor/SB; EBs were found to be round, uniform in the presence of Dor/SB.

 For successful generation of NPCs, it is crucial to start with pristine, undifferentiated iPSC cultures. IPSC colonies should be densely packed show low nucleus to cytoplasma ratios and have discrete borders and no differentiation along the peripheries and/or in the centers of the colonies. In this protocol, we found that 2 mm diameter sized colonies yield the best results for neural rosette formation (Figure 2(a)).

### 3.2. Combination of Dorsomorphin and SB431542 Improved Neural Induction

**Figure 3:**
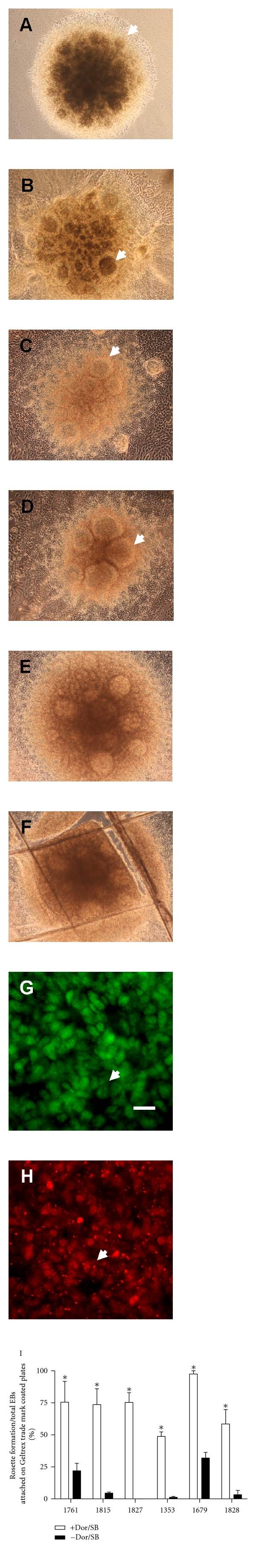
Representative images of neural rosettes at different stages (A–D). Neural rosettes after Dor/SB treatment at days 7–12, stage III of the differentiation protocol (5x magnification). Arrows indicate boundary lines in the early and late stages of rosettes. (E) and (F) Manual dissection of rosettes using scalpel are illustrated before (E) and after (F) cutting. (G and H) Arrows indicate the positions of neural rosettes that were immunoreactive with Pax6 (green) (E) and Sox1 (red) (F). Scale bar represents 20 *μ*m. (i) There is a significant difference in neural rosette formation in all control and PD-specific EBs treated with Dor/SM compared to those with no Dor/SB treatment during stage II (*P < 0.01). Data are presented as mean + standard error of the mean (SEM) compared to the controls (*n* = 3, except 1679, *n* = 2). *P* value of each study was assessed by one-way ANOVA along with Newman-Keuls post-*hoc* analysis.

IPSC colonies were enzymatically treated with collagenase. After detachment, half of the colonies in a dish were exposed to 5 *μ*M Dor/10 *μ*M SB. The other half was left untreated. The colonies were then cultured for 4 days in EB media with or without Dor/SB. EBs cultured in EB media alone showed loose, less compact, and irregular shapes (Figure 2(b)) while the majority of EBs treated with Dor/SB demonstrated compact, solid and round shaped aggregates and had an average size of 350 *μ*m in diameter (Figure 2(b)). On day 4, media was changed to NIM media containing N2 media which was freshly made of different individual components. None of the commercially available N2 supplements showed consistent results (data not shown). EBs were then plated onto Geltrex-coated culture dishes on day 6. During days 6–10, neural rosettes were detected by their characteristic morphology of radially arranged cells (Figures 3(A)–3(D)). In the early stages of NIM incubation (approximately days 8–10), neural rosettes showed darker centers of “flower-shaped” structures with indiscrete boundary lines (Figures 3(A) and 3(B)). In the latter incubation with NIM, “flower-shaped” morphologies were more distinct and edges more clearly defined, shown in Figures 3(C) and 3(D). Dissected rosettes (Figures 3(E) and 3(F)) that are replated and manually isolated a second time generate NPC populations of higher purity.

We evaluated neural differentiation of EBs on day 10 (Figure 1(b)) via immunocytochemistry. Neural markers Pax6 and Sox1 were used as well as the pluripotent cell marker Oct4. Pax6 and Sox1 showed positive staining in attached EB (Figures 3(G) and 3(H)), however, Oct4 showed no immunoreactivity (data not shown). At the same time point, the percentage of neural rosettes formed with and without addition of Dor/SB was quantified by manually counting the colonies containing neural rosettes divided by total colonies attached on the culture dish (Figures 1(b) and 3(I)). Without Dor/SB, we observed low rosette formation between 0% and 31.9%, and we were not able to derive expandable NPCs. The combination of Dor/SB, on the other hand, increased the neural rosette formation substantially to 48% to 97.5% of EBs in both control and PD-specific cell lines. Overall, we did not notice a difference in the efficiency of rosette formation between PD lines and control lines.

**Figure 4:**
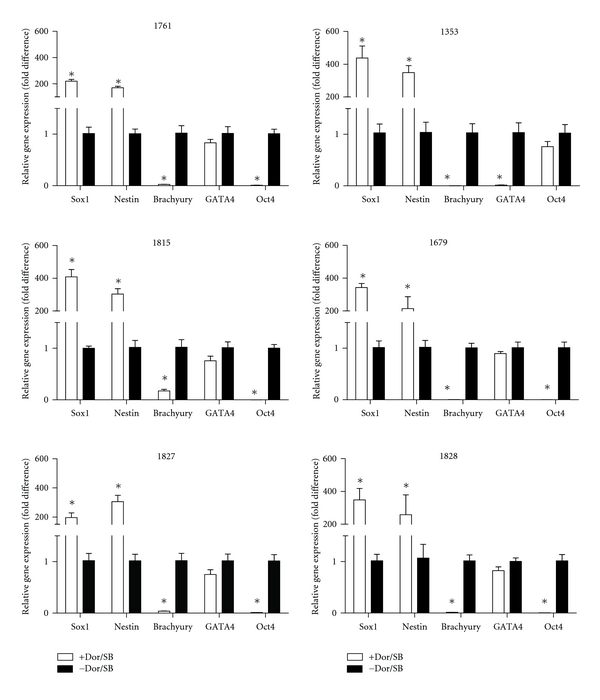
Quantitative gene expression analysis of PD and control lines with and without Dor/SB. Expression levels of neuroectoderm (Sox1 and Nestin), mesoderm (Brachyury), endoderm (GATA4), and pluripotent markers (Oct4) were assessed by quantitative PCR. The *y*-axis represents means + SEM of relative expression levels of each gene in the EBs with Dor/SB relative to no Dor/SB treatment (*P < 0.01). Left panel depicts lines from healthy donors; right panels depicts cell lines derived from patients with PD. Data are presented as mean + SEM compared to the controls (*n* = 3, except 1679, *n* = 2). *P-*value of each study was assessed by one-way ANOVA along with Newman-Keuls post-*hoc* analysis.

At day 6, we performed gene expression analysis of multiple markers in attached EBs. In all six lines we studied neuroectodermal markers Sox1 and Nestin, mesodermal marker Brachyury, endodermal marker GATA4 and pluripotent marker Oct4. Surprisingly, there was a striking difference of >150-fold in the gene expressions of neuroectodermal markers Sox1 and Nestin in Dor-/SB-treated EBs compared to EBs without small molecules (Figure 4). This suggests that the two small molecules very efficiently modulate the SMAD signaling pathway leading to this enormous increase in neuroectodermal markers. This increase was consistent in all six iPSC lines tested, and differences in neuronal differentiation were not observed between patient and control lines. Endo and mesodermal markers GATA4 and Brachyury as well as pluripotency marker Oct 4 were all lower compared to the untreated, normalized NPC lines.

**Figure 5:**
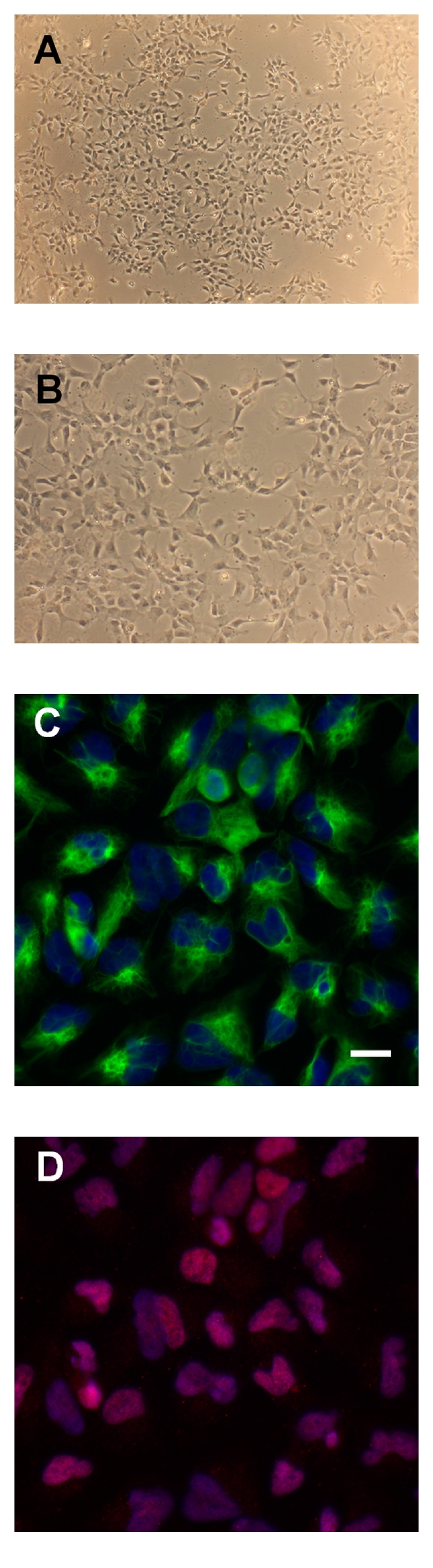
Characterization of NPCs. (A) and (B) NPC morphology was observed under phase-contrast microscopy with 5x (A and 10x (B) magnification. (C and D) NPCs expressed Nestin (green) (C) and Sox1 (red). (D) Nuclei were counterstained with DAPI (blue). Scale bar represents 20 *μ*m.

Neural rosettes were manually cut and replated as pieces to produce a population of NPCs of higher purity. Rosettes were manually isolated once again, collected, enzymatically treated with Accutase, and plated and expanded in NPC media. Manual passaging and expansion of NPCs still yielded approximately 10% undifferentiated Oct4-positive cells in NPC cultures, which upon further expansion showed iPSC morphology (data not shown). Therefore, we used magnetic bead sorting with a neural cell adhesion molecule antibody against polysialic acid neural cell adhesion molecule (PSA-NCAM or CD56) (Figures 5(A) and 5(B)). We observed an approximately 20% cell loss after magnetic bead sorting. We characterized NPCs after sorting by immunocytochemistry with defined markers Nestin and Sox1. We detected >90% Nestin and Sox1 immunoreactive NPCs in all iPSC cell lines taken through this protocol (Figures 5(C) and 5(D)). NPCs were readily expandable at a passaging ratio of 1 : 2 to 1 : 3 with Accutase. Cultures grew well when media was prepared freshly every 2 to 3 days and B27 added freshly to NPC media, before media changes. We expanded NPC cultures for >15 passages after derivation and did not observe any changes in morphology or expression of Nestin and Sox1.

 With this new approach for neural induction using small molecules, we have dramatically increased reproducibility and efficiency of neural rosette stage/NPC generation. This is invaluable when using patient-derived iPSCs for disease modeling, which may have an intrinsic disadvantage in culture when carrying potential disease-related deficiencies. Since NPCs can be easily expanded, this could become a suitable cell type for high throughput screening where a very large number of starting material is needed.

### 3.3. Substitution of Small Molecules Purmorphamine or Smoothened Agonist for Sonic Hedgehog Had Similar Effects on Neuronal Maturation

We investigated the substitution of sonic hedgehog (Shh) for small molecules purmorphamine (Pur) or smoothened agonist (SAG) during dopaminergic maturation. These chemicals that are considerably less expensive, have minimal lot-to-lot variabilities, and have longer shelf-life compared to recombinant proteins.

**Figure 6:**
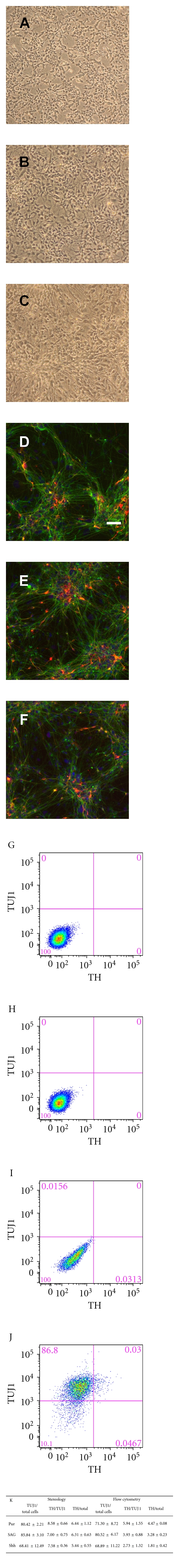
Analysis of number of TH+ and TUJ+ neurons (A)–(C). Image under phase-contrast microscopy showed plating density of NPCs at day 1 in DA1 media (5x magnification) (A), cell morphology at day 12 (arrows indicate the formation of processes) (B) 10x magnification) and neurons at day 30 during dopaminergic maturation (C) 10x magnification). (D)–(F), Neurons treated with Pur (D), SAG (E) and Shh (F) were characterized by immunostaining with TUJ1 (green) and TH (red) and counterstained with DAPI (blue). Scale bar represents 200 *μ*m. (G)–(J) Flow cytometry: No TUJ1 and TH immunoreactive NPCs were detected (H), which is comparable to unstained NPCs (G). In contrast to the population of unstained differentiated neurons (I), differentiated cells showed positive immunoreactivity to TUJ1 and TH (j). (k) Table illustrates percentage of TUJ1 relative to total cells, TH immunoreactive neurons relative to TUJ1 immunoreactive neurons, and also relative to total cells quantified using stereology and flow cytometry in control NPCs after dopaminergic maturation.

 For final dopaminergic maturation, we used a 2-step approach. For the first ten days, we cultured NPCs in FGF8 and tested two small molecules SAG and Pur as substitutes for Shh in control and patient cell lines. At day 1 in DA1 media, the plating density of the NPCs should be approximately 60% to 70% (Figure 6(A)). During this 10-day protocol, cells were split at 100% confluency using Accutase and replated at a cell density of approximately 80%. When cells were plated at a lower cell density (<50%), we observed remarkable cell death and low rates of cell attachment. After ten days, we switched to Neurobasal media supplemented with BDNF, GDNF, dcAMP, and B27 every second day, but added B27 daily preventing cell death. Cells were split until they began growing out processes (Figure 6(B)). After day 30 of dopaminergic maturation, cells were fixed, immunostained with TUJ1 and TH, and counterstained with DAPI (Figures 6(C)–6(F)).

 To measure the efficiency of the neuronal differentiation, we evaluated the percentage of TH and TUJ1 expressing neurons relative to total cells using two approaches: stereology with systematic random sampling and flow cytometry. Flow cytometry was employed to minimize bias. The challenges of accurate counting of these cultures are the dense “patches” of neurons and the majority of TH immunoreactive neurons localized in these “patches” [37].

In the scatter plots for flow cytometry, (Figures 6(G)–6(J)), undifferentiated NPCs did not show immunoreactivity for TUJ1 and TH (Figure 6(H)) and had a similar pattern in the scatter plot to unstained NPCs (Figure 6(G)). Differentiated neurons were immunoreactive for TUJ1 and TH (Figure 6(I)) and were compared to the total number of unstained differentiated neurons (Figure 6(J)).

 Both approaches, stereology and flow cytometry, showed no significant differences among the three different components Shh, SAG, or Pur used in dopaminergic differentiation in terms of the ratio of TUJ1/total, TH/TuJ1, and TH/total cells (Figure 6(K)). Data from flow cytometry was slightly lower than those from the stereological approach. We suspect that we lost neurons during the handling process such as dissociation and passaging through a cell strainer to filter clumps from cell suspension before flow cytometry was performed.

 Some studies have shown that with an extension of culturing time by up to 60 days, more neurons convert to TH-positive as well as become electrophysiologically mature [37, 38]. Other studies showed a higher percentage of TH-positive neurons, however, different quantification approaches may have introduced bias toward a higher percentage of neuronal yields.

## 4. Discussion: Small Molecules for Efficient Neuronal Differentiation

Over the last few years, there has been an enormous push to optimize differentiation protocols with different small molecules and screens to identify new factors that would modulate and improve neuronal differentiation and maturation.

 Other small molecules and compounds have been identified for the enhancement of neuronal differentiation. Glycogen synthase kinase-3 (GSK-3) inhibitors such as kenpaullone or SB-216763 have been shown to positively impact the neuronal differentiation of neural progenitor cells without changing cell cycle exit or cell survival [39]. Furthermore, GSK-3 inhibitors showed protection against excitotoxicity, mediated by NMDA and non-NMDA receptor agonists, in cultured rat primary cerebellar granule neuronal cultures from the cerebellum and hippocampus [40].

(+)-Cholesten-3-one but not cholesterol has been shown to effectively promote the activity of the TH promoter. (+)-Cholesten-3-one has also been shown to induce differentiation of neuroprogenitors into dopaminergic neurons monitored by expression of TH, dopamine transporter, dopa decarboxylase, and higher levels of dopamine secretion [41].

Neurosteroids are thought to affect neuronal survival, neurite outgrowth, and neurogenesis both *in vivo* and *in vitro* [42], that is, progesterone [43] and estradiol [44]. Progesterone added at the neural proliferation stage increased the number of dopaminergic neurons, whereas progesterone added during final differentiation did not induce significant changes in the number of dopaminergic neurons generated. Interestingly, this effect was not mediated by the activation of progesterone receptors because RU 486 did not block the effects of progesterone on dopaminergic differentiation [43]. It has also been shown that estradiol can increase the generation of dopaminergic precursors expressing Lmx1a and can induce formation of a higher percentage of mature dopaminergic neurons [44].

In addition, polyunsaturated fatty acids such as arachidonic acid (ARA) and docosahexaenoic acid (DHA) have been shown to have critical roles in brain development and function and can promote neurogenesis [45]. Specifically, DHA, a ligand for the RXR/Nurr1 heterodimer, can activate the Nurr1 gene in iPSCs. It has been shown that DHA facilitates iPSC differentiation into TH-positive neurons *in vitro* as well as *in vivo* [46].

 Through a peptide library screen a novel small synthetic peptide Cripto BP was discovered to block Cripto, a glycosylphosphatidylinositol-anchored coreceptor. It has been shown that this receptor binds Nodal and the ALK-4 receptors and promotes cardiac differentiation. The deletion or inhibition of Cripto leads to a promotion of neuronal and midbrain differentiation of mouse embryonic stem cells. The synthetic peptide Cripto BP can mimic this effect [47].

## 5. Conclusion

Small molecules can enhance various steps of neuronal differentiation into dopaminergic neurons and can replace expensive recombinant proteins that were initially used in the pioneering protocols. However, there is still a need for improvement of differentiation protocols that increase the number and region-specificity of mature region specific dopaminergic neurons, but selective inhibitors and other small molecules might change the field and reduce the cost.
